# Surgical vs. non-surgical management of displaced type-2 odontoid fractures in patients aged 75 years and older: study protocol for a randomised controlled trial

**DOI:** 10.1186/s13063-018-2690-8

**Published:** 2018-08-22

**Authors:** Anna-Lena Robinson, Gregor Schmeiser, Yohan Robinson, Claes Olerud

**Affiliations:** 1 Department of Surgical Sciences, Uppsala University Hospital, Uppsala, Sweden; 2 Stockholm Spine Center, Stockholm, Sweden; 3 Schön Clinic Hamburg Eilbek, Hamburg, Germany; 4 Dept. of Research and Development, Armed Forces Centre for Defence Medicine, Västra Frölunda, Gothenburg, Sweden; 5 Stockholm Spine Center, Löwenströmska Hospital, 194 89 Stockholm, Upplands Väsby Sweden

**Keywords:** Odontoid fractures, Elderly, Osteoporosis, Spinal fractures, Surgical treatment

## Abstract

**Background:**

Displaced odontoid fractures in the elderly are treated non-surgically with a cervical collar or surgically with C1–C2 fusion. Due to the paucity of evidence, the treatment decision is often left to the discretion of the expert surgeon.

**Methods:**

The Uppsala Study on Odontoid Fracture Treatment (USOFT) is a multicentre, open-label, randomised controlled superiority trial evaluating the clinical superiority of the surgical treatment of type-2 odontoid fractures, with a 1-year Neck Disability Index (NDI) as the primary endpoint. Fifty consecutive patients aged ≥ 75 years, with displaced type-2 odontoid fracture, are randomised to non-surgical or surgical treatment. Excluded are patients with an American Society of Anaesthesiologists (ASA) score ≥ 4, dementia nursing care or anatomical cervical anomalies.

The minimal clinically important difference of the NDI is 3.5 points. A minimum of 16 patients are needed in each group to test the superiority with 80% power. By considering a 1-year mortality forecast of 29%, up to 25 participants are recruited in each group.

The non-surgical group is fitted with a rigid cervical collar for 12 weeks. The surgical group is treated with a posterior C1–C2 fusion. All participants are monitored with regard to the NDI, EuroQol score (EQ-5D), socio-demographics and computed tomography (CT) at the time of injury, at 6 weeks, 3 months and 12 months. At 12 months, a dynamic radiographical investigation of upper cervical stability is performed.

The secondary endpoints are: EQ-5D score, activities of daily living (ADL), bony union, upper cervical stability and mortality.

**Discussion:**

USOFT is the first randomised controlled trial comparing non-surgical and surgical management of type-2 odontoid fractures in the elderly. Using the NDI and EQ-5D as endpoints, future value-based decisions may consider quality-adjusted life years gained. Major limitations are (1) the allocation bias of the open-label study design, (2) that only higher training levels of all core specialties of spine surgery are included in the surgical treatment arm and (3) that only one type of surgical stabilisation is investigated (posterior C1–C2 fusion), while other methods are not included in this study.

**Trial registration:**

ClinicalTrials.gov, NCT02789774. Registered retrospectively on 25 August 2015.

**Electronic supplementary material:**

The online version of this article (10.1186/s13063-018-2690-8) contains supplementary material, which is available to authorized users.

## Background

### Background and rationale

Unstable injuries to the upper cervical spine are a hazard to every elderly person, since associated dysphagia and respiratory restrictions are potential deadly complications [1]. The most common upper cervical fracture in elderly people aged ≥ 70 years is the type-2 odontoid fracture, with an incidence of 15.6 per 100,000 person-years in 2014 [2]. As with other osseous injuries in the elderly, the occurrence of odontoid fractures has been related to fragility, osteoporosis and falls [3]. The mortality of odontoid fractures, meanwhile, is strongly related to age, gender, comorbidity and type of treatment [4].

The treatment of displaced type-2 odontoid fractures in the elderly varies from external orthosis treatment with a rigid collar to surgery, including anterior screw osteosynthesis and posterior C1–C2 fusion. Some earlier non-randomised studies have shown no significant difference between surgical treatment or non-surgical treatment regarding functional outcome (NDI, Neck Disability Index) or functional outcome score), pain and patient satisfaction [5, 6] the AOSpine North America Geriatric Odontoid Fracture (GOF) study has found significantly better outcomes measuring the NDI and the SF-36 Bodily Pain dimension comparing non-surgically and surgically treated patients [7]. Despite the fatal implications of inaccurate treatment, the most recent treatment recommendations vary widely, while surgeon adherence is highly dependent on regional differences [8]. There is a trend towards non-surgical treatment in Sweden; the fear of overtreatment could be a contributing factor. Patient comorbidity could be another explanation for physicians’ tendency to use a cervical collar in the belief that this will avoid further damage. It could also be the tradition of the clinic that determines what treatment the patient should receive.

### Evidence

On 30 October 2017, a search in PubMed was performed with the search term “odontoid[Title] OR dens[Title] AND fracture[title]”. Of the 321 search results published between 1985 and 2017, only two prospective study were identified [7, 9, 10]. In the first prospective observational study, on percutaneous transarticular atlantoaxial screw fixation, 20 consecutive, multimorbid patients aged > 65 years with a type-2 odontoid fracture were included and followed for a minimum of 18 months [9]. The results from the study showed 88% healing of the fracture and 15% mortality within 3 months. In the other study 159 patients with a type-2 odontoid fracture were included in a multicentre prospective study, comparing surgical (*n* = 101) and non-surgical (*n* = 58) treatment. Treatment choice was determined by the physician and/or the patient. The subjects were followed at 6 and 12 months with outcome measures, including the Neck Disability Index (NDI) and Short Form-36v2 (SF-36v2). They found that the functional outcome was significantly better in the surgically treated patients [7].

There were three ongoing prospective studies (including USOFT) registered at the ClinicalTrials.gov database (status: recruiting, NCT02281994, NCT02800278, NCT02789774). Meta-analyses of the survival from type-2 odontoid fractures among the elderly aged ≥ 65 years report better survival for patients who are treated surgically (Hazard ratio (HR) = 0.64) [4], while this seems to diminish if only those aged ≥ 80 years are included [11]. Due to the retrospective nature of most published studies, considerable reporting and selection bias must be assumed, meaning that the strength of current treatment recommendations is questionable.

### Implications of this study

Previously published and ongoing studies are focussed on non-union and mortality in relation to different treatment modalities. Nowadays, value-based care requires health-related quality of life (HRQoL) measures to influence health policy and decision-makers. By studying HRQoL and the function of patients with type-2 odontoid fractures, while comparing non-surgical treatment and the most common surgical treatment (posterior C1–C2 fusion), we can address the cost-effectiveness of the treatment options.

## Methods

### Study aims and objectives

#### Primary objective

The primary objective is to compare neck disability at 1 year after non-surgical treatment with surgical treatment of displaced type-2 odontoid fractures.

#### Secondary objective

The secondary objectives are to compare the cost-effectiveness, the mortality, the HRQoL and the bony union of non-surgical with surgical treatment after 1 year.

#### Trial design

The Uppsala Study on Odontoid Fracture Treatment (USOFT) is a multicentre, open-label, randomised controlled superiority trial. The protocol follows the Standard Protocol Items: Recommendations for Interventional Trials (SPIRIT) Statement for clinical trial reporting (Additional file 1) [12].

### Study setting

The USOFT is being conducted in five departments of orthopaedic surgery in three university hospitals (Uppsala University Hospital, Malmö University Hospital and Karolinska University Hospital Stockholm) in Sweden. Participating university hospitals are level 1 trauma centres. All treating spine surgeons are orthopaedic or neurosurgical specialists with different levels of clinical experience in treating upper cervical spinal conditions.

### Eligibility criteria

#### Inclusion criteria

Patients are eligible for inclusion in this study if they meet all of the following criteria:

- Acute displaced odontoid fracture, type 2 [13]
  - ▪ Displacement is measured on a multiplanar reconstruction of a cervical spine CT scan and defined as a 5-mm anterior translator displacement, any posterior translator displacement or 10° of angulation [14–17]
- Age: 75 years or older

#### Exclusion criteria

Patients are not included in this study if they meet one or more of the following criteria:

- ASA class 4 or higher [18, 19]
- Severe dementia
  - ▪ Defined as being admitted to a nursing home or hospital because of dementia
- Anatomical anomalies (i.e. occipitocervical assimilation)
- Injuries (i.e. spinal cord injury (SCI) or severe dislocation threatening SCI) that mandate surgery

### Interventions

#### Concomitant treatment in both groups

- Collar in emergency department (Stifneck, Laerdal Medical, Stockholm Sweden)
- Pain medication (bolus: 2–5 mg morphine by the intravenous (IV) route)
- Diagnostic workup
- Baseline data collection according to Fig. 2
- Expertise of participating physicians: beginner (residents) to expert (consultant)
- Follow-up after 6 weeks, 3 months and 1 year, according to Fig. 2

#### Non-surgical treatment

The patient will be provided with a well-fitted and rigid collar, such as the Philadelphia collar (Ossur, Sollentuna, Sweden) or the Aspen collar (Aspen Medical Collars, Irvine, CA, USA). The collar will be worn 24 h a day for 12 weeks. The collar may be removed for short periods of time for personal hygiene when the patient is supine in bed.

CT scans will be repeated after 1 week, 6 weeks and 3 months. In case of increasing fracture displacement, or symptomatic non-union, the fracture will be treated surgically with a posterior C1–C2 fusion. The crossover will be documented, but all data will be analysed according to the intention-to-treat (ITT) principle.

#### Surgical treatment

The patient will be surgically treated with an instrumented posterior C1–C2 fusion, including an autologous iliac crest bone graft. The primary fixation technique involves transarticular C1–C2 screws, according to Magerl, and the C1 claw device [20]. In case of anatomical aberrations or a concomitant C1 arch fracture, other techniques may be used: e.g., C1 screws, according to Goel-Harms [21, 22], or translaminar C2 screws, according to Wright [23].

### Study outcome measures

#### Primary outcome measure

The primary outcome variable is the difference in the Neck Disability Index (NDI) at baseline and 1 year after injury [24, 25]. Both comparing differences within the groups (baseline vs. NDI at 1 year), but also differences between the groups. The same evaluation will be made at 6 weeks and 3 months.

#### Secondary outcome measures

- EQ-5D score
- Katz ADL (Activities of Daily Living score) at baseline and at 1 year
- Visual Analogous Scale (VAS) at baseline and at 1 year
- Mortality
- Serious adverse events (including death) during the first year
- Radiographically demonstrable healing on CT after 1 year
- Upper cervical stability on dynamic flexion-extension radiographs of the cervical spine after 1 year

#### Subgroup analysis

- Socio-demographics
  - ▪ Age
  - ▪ Gender (male/female)
  - ▪ Body Mass Index (BMI)
  - ▪ American Society of Anaesthesiologists (ASA) class
  - ▪ Smoking (yes/no)
- Type-2 odontoid fracture subgroup
  - ▪ Grauer classification [15]
- Type of posterior C1–C2 fusion
  - ▪ Goel-Harms technique or Magerl-Atlas claw technique
- Osteoporosis (dual energy x-ray absorptiometry (DXA) score)

### Participant timeline

**Fig. 1:**
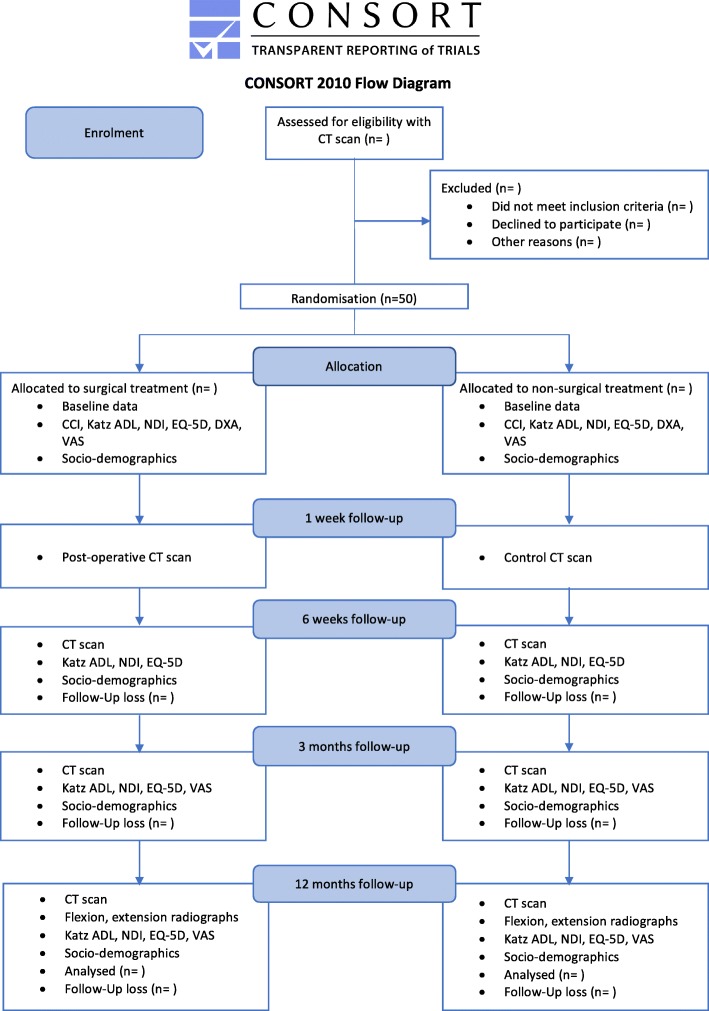
Consolidated Standards of Reporting Trials (CONSORT) inclusion flow diagram

**Fig. 2:**
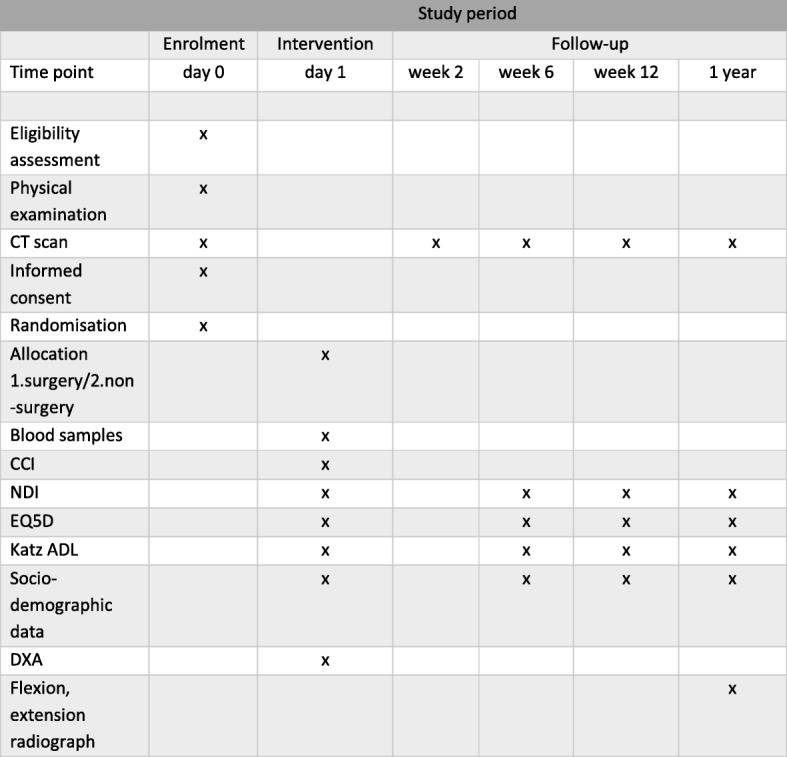
Participant timeline. *CT* computed tomography, *CCI* charlson comorbidity index, *EQ-5D* euroQol, *NDI* neck disability index, katz *ADL* activities in daily life according to katz, *DXA* double x-ray absorptiometry

The Consolidated Standards of Reporting Trials (CONSORT) inclusion flow diagram is presented in Fig. 1 [26]. The USOFT participant timeline is described in Fig. 2.

### Recruitment

The history and physical examinations of all patients scheduled for surgery are screened preoperatively for predictors of difficult airways. Patient recruitment is conducted by a physician, while patient-reported follow-up is controlled by a qualified study nurse.

### Assignment of intervention

#### Allocation

When a patient with an eligible type-2 odontoid fracture seeks care at the hospital, the surgeon in charge of emergency care will contact the study group who inform the patient about the study and obtains their informed consent (Additional file 2). If the patient is unable to give informed consent, a close relative will be asked to give permission. Allocation is randomised using a sequentially numbered, opaque, sealed envelope (SNOSE) technique [27]. For obvious reasons, this study is not blinded.

If the patient/relative chooses not to participate in the USOFT, the patient is treated with a rigid collar, i.e. the present treatment at the department. Socio-demographic and clinical data will be collected. After acute treatment and mobilisation, the patient will be discharged to a suitable level of care in a normal way.

#### Sequence generation

Simple randomisation will be used to generate the allocation sequence. Randomisation is concealed using a SNOSE technique. Once a patient has given written consent, the next envelope according to the numbered sequence is opened and the patient is treated according to the allocated treatment.

#### Blinding

Blinding is not possible.

### Study instruments

#### Neck Disability Index

The NDI quantifies neck-related disability on a score from 0 (no disability) to 50 (maximum disability) [24]. The results are then transformed into percentages (50 point = 100% disability). As many of the patients aged ≥ 75 years no longer drive a car, question 8, which concerns driving a car, may be excluded, resulting in a maximum score of 45. The minimal clinically important difference (MCID) of the NDI is 3.5 points, with a standard deviation (SD) of 3.5 points [28]. The modified Swedish version of the NDI has been tested, showing good validity, sensitivity, test-retest reliability and specificity [25].

#### EuroQol health-related quality of life questionnaire (EQ-5D)

EQ-5D is a standardised self-rating instrument developed by the EuroQol group as a measure of HRQoL and translated into Swedish [29]. The EQ-5D consists of a descriptive system and the EQ Visual Analogue Scale (VAS). In the questionnaire, the study participant classifies their health in five dimensions (mobility, hygiene, main activities, pains/inconvenience, worry/mood) in one of three degrees (no problems = 1, moderate problems = 2, difficult problems = 3). The five questions in the health questionnaire make it possible to determine 243 different health conditions. The value may be negative, due to the conversion system, and varies between 1 and − 0.594. To enable health economic analyses (i.e. quality-adjusted life-years (QALY) calculations), each of the 243 health conditions has a quality of life tariff linked to it. In Sweden, we use the English tariff [30, 31].

EQ VAS (self-assessed health): the individual estimates a value for their current state of health on a scale graded from 0 to 100.

#### Katz ADL Index

The Katz ADL Index comprises six basic functions: bathing, dressing, toileting, transfer, continence and feeding. It provides an objective method of classifying and describing an individual’s health needs and outcomes. For each activity (six in total), there is a question. Each question has two possible answers: independent or dependent [32]. The Katz ADL Index is an ordinal scale ranging from A to G, where A means that the patient is totally independent of any assisting help. F is considered to be an indicator of a dependent patient. There is no ‘cut-off’ limit. The score will be transformed into a nominal scale (1–7) where A = 1, B = 2, etc.

#### Computer tomography

CT is the most reliable radiographic tool to classify and evaluate healing in relation to type-2 odontoid fractures [33]. Bony union is assessed by a radiologist, independently from the study. Healing is defined as ‘not visible fracture line’ or ‘bone bridging in fusion’.

#### Extension flexion plain radiograph

Dynamic flexion-extension radiographs are used to assess the functional stability – the healing – of a type-2 odontoid fracture [34]. Instability is defined by a > 3.5-mm listhesis and/or a relative angulation of > 11° [35].

#### Dual energy X-ray absorptiometry

Osteoporosis will be evaluated with DXA of the hip and lumbar spine [36].

#### Other tests

- Blood samples (including haemoglobin, electrolytes)
- Socio-demographics, living situation (1. home, 2. nursing home, 3. hospital)
- Charlson Comorbidity Index [37, 38]
- VAS for pain (0 to 100)

### Data collection, management and analysis

#### Data collection and management

The USOFT baseline data are recorded in a paper-based clinical record folder. Prior to inclusion, the data from each patient are collected by a spinal surgeon or a dedicated study nurse. All physician-reported outcome measurements are recorded during and after the evaluation in the folder. Patient-reported outcome measurements are recorded in the Clinical Trials extension of the Swedish Spine Registry (SweSpine) [39]. Any protocol deviations are recorded either in the case file or in the medical records; a clinical study nurse ensures that all protocol deviations and adverse events are recorded in the database.

Every allocated subject will be coded with a specific patient number. The study data will be transferred to a pre-made computer-based table (Microsoft Excel, V.15.32, Microsoft, Redmond, WA, USA) and the Clinical Trials extension of SweSpine [39]. The completed files will be stored securely in the clinical research unit for the next 15 years.

#### Access to data

Data safety, data quality and statistical analysis will be managed by the principal investigator who is responsible for notifying any issues that may arise during the USOFT. Data are collected and stored according to Good Clinical Practice guidelines and are available to all participating study sites. Any data safety issue occurring during the clinical trial will be reported to the principal investigator.

### Statistics

For statistical analysis, R version 3.4.1 (R Foundation for Statistical Computing, Vienna, Austria) will be used. The results will be presented according to the CONSORT Statement for non-pharmacological interventions [26]. The analysis will be performed by intention-to-treat (ITT), and sensitivity analyses will include a per-protocol analysis.

Missing values will be imputed using multiple imputation by use of chained equations as implemented in the R package “mice” with 20 iterations [40]. Details of the statistical analysis are listed in Table 1.

**Table 1:** Variables, measures and methods of analysis

Variable/Outcomes	Hypothesis	Outcome measures	Methods of analysis
*Baseline data:*	There is no difference between the two groups	Gender, age, Katz ADL, CCI, nursing home/hospitalisation, smoking status	Absolute numbers, percentages for categorical variables and the minimum, maximum, mean, SD and quartiles for quantitative variables
*Primary:*			
Function	There is a clinically important difference between the two groups, with improvement comparing baseline data with data from 6 weeks, 12 weeks, and 1 year. Surgical treatment is hypothesised to be superior	NDI (0–100%) [continuous]	Student *t* test, chi-square, Mann-Whitney, Fisher’s exact test. Time-dependent differences between AUC
*Secondary:*			
Health-related quality of life	There is a clinically important difference between the two groups	EQ-5D [continuous]	Student *t* test, chi-square, Mann-Whitney, Fisher’s exact test Time-dependent differences between AUC
Pain	There is a clinically important difference between the two groups	VAS (0–100) [continuous]	Students *t* test, chi-square, Mann-Whitney, Fisher exact test Time-dependent differences between AUC
Non-union	The non-union rate is lower in the surgical group	Bone bridge in CT [binary], mobility on extension-flexion radiographs [binary],	Chi-square test
Mortality	The survival is greater in the surgical group	all-cause mortality [binary], time to death [continuous, censored]	Kaplan-Meier analysis, Cox regression models, additional subdistribution hazards approach
Osteoporosis	There is no difference between the two groups	The bone density is > 2.5 standard deviations below normal DXA T-score [binary]	Chi-square
*Subgroup analysis:*			
septuagenarians vs. octogenarians vs. nonagenarians	Treatment effect is diminished in nonagenarians		
Goel-Harms technique vs. Magerl technique	There is no difference between the two groups		
Male vs. female	There is no difference between the two groups		
*Sensitivity analysis:*			
Per-protocol analysis		All outcomes	Students *t* test, chi-square, Mann-Whitney, Fisher exact test
Adjusting for baseline covariates	All outcomes		Uni-and multivariate adjusted logistic regression and Cox proportional hazard models
Adjusting for mortality	All outcomes		subdistribution hazards approach

*CT* computed tomography*, NDI* Neck Disability Index, *EQ-5D* EuroQol, *VAS* Visual Analogue Scale, *CCI* Charlson Comorbidity Index, *Katz ADL* Activities of Daily Living score according to Katz, *DXA* double x-ray absorptiometry, *AUC* area under the curve

#### Description of the patient groups at baseline

The baseline features of the patients will be described with descriptive statistics using absolute numbers (*n*) and percentages for categorical variables and the minimum, maximum, mean, SD and quartiles for quantitative variables. The number of patients crossing over to surgical treatment or dropping out from follow-up will be documented.

#### Analysis of the primary outcome

Multiple regression analysis of subgroup factors will allow for the determination of important factors affecting the NDI. The differences will be considered statistically significant if the *p* value is less than 0.05.

### Analysis of the secondary outcomes

#### EQ-5D

HRQoL will be evaluated with the EQ-5D by comparing the results from the two groups, using the independent *t* test, the Mann-Whitney *U* test or the Fisher exact test.

The differences will be considered statistically significant if the *p* value is less than 0.05.

### Non-union

A chi-squared test will be used to compare the non-union rate between the two groups.

### VAS

The VAS will be evaluated using the *t* test, the Mann-Whitney *U* test or Fisher’s exact test.

### Survival

The Kaplan-Meier method will be used for the determination of the non-surgical and surgical treatment mean survival at 1 year. Proportional survival differences according to treatment will be tested with the chi-squared test. With the Cox proportional hazards regression method, covariates contributing to survival will be entered in univariate and multivariate models, if the hazard ratio presents with a 95% confidence interval (CI).

#### Subgroup analysis

We will perform a separate analysis of participant socio-demographics, type-2 odontoid fracture subgroup, the specific type of surgery (categorical: Magerl-Atlas claw or Goel-Harms) and osteoporosis status (DXA score).

### Sample size

Up to 50 participants will be included in the study, based on the following calculation of sample size:

The MCID of the NDI is 3.5 points (7%) and the SD is 3.5 points [28]. Thus, 16 participants are needed in each group to reach 80% power with a two-sided significance level of *p* < 0.05 in a (clinically important) superiority study design. Adjusting for 1-year mortality among those aged ≥ 70 years with a non-surgical axis fracture treatment of 29% (Robinson AL et al. Spine J. 2018; in press), eight additional participants must be recruited in each group. A minimum of 24 subjects must be included in each group.

### Monitoring

#### Data monitoring

An interim analysis will be performed on the primary endpoint (NDI improvement) when 30 patients have been randomised and completed the 1-year follow-up. The interim analysis will be performed by a statistician. The statistician will report to the R&D Council of the Orthopaedic Department at Uppsala University Hospital (FoUU rådet). The R&D Council will have unblinded access to all data. The council, which will decide on the continuation of the trial, will report to the Central Ethics Committee if the trial is stopped prematurely [41]. The decision regarding study discontinuation follows the Haybittle-Peto stopping rule: if treatment effects cause an NDI improvement difference between surgical and non-surgical groups with *p* < 0.001 in the interim analysis, the trial will be stopped [42].

#### Harm

If the attending physician suspects a serious adverse event (SAE), the patient’s follow-up will not be discontinued. An SAE is considered if it results in the following outcomes: in-hospital death, life-threatening event or neurological worsening. In case of severe adverse advents, the investigator in charge of the study will inform the R&D Council. The council will then decide on whether to continue the trial, reporting to the Central Ethics Committee if the trial is stopped prematurely.

#### Auditing

The SweSpine board (governed by the Swedish Society of Spinal Surgeons) reviews the patient-reported outcome measure forms and clinical data at regular intervals.

### Ethics and dissemination

#### Research ethics approval

This study is conducted in compliance with the current version of the Declaration of Helsinki. The research project was approved by the Uppsala Regional Ethics Committee on 18 May 2011 (registration no. 2011/068).

#### Protocol amendments

Modifications to the protocol require approval by the Regional Ethics Committees, will be registered with ClinicalTrials.gov, and will be communicated to the participating university hospitals.

#### Consent or assent

Prior to the trial, patients must consent orally and in writing after the possible consequences of the clinical study are explained in an understandable way. All documents must be written in Swedish. If the patient is unable to give informed consent, a close relative will be asked to take the decision instead. The patient will receive a copy of the signed patient information. A patient may withdraw from the study at any time if they are unwilling to continue on the trial. In this case, the data from a patient who requests full withdrawal will not be considered in the data analysis.

#### Confidentiality

All original documents will be kept in the clinical research unit for the next 15 years. The study data will be handled in line with by the European Directive 95/46/EC on data protection. All original records will be kept on file at the trial sites or coordinating data managing centre for 10 years. The electronic clinical trial database in SweSpine will be kept on file for at least 10 years.

#### Access to data

The principal investigators have full access to the final datasets. There are no contractual agreements that limit such access for investigators.

#### Dissemination policy

The study results will be published in peer-reviewed medical journals, and communicated at medical conferences. The original principal investigators AR, YR and CO will appear as co-authors on all publications based on results from this study cohort. The participant-level dataset and the statistical code will not be publicly available and remain with the principal investigators.

## Discussion

This is the first randomised controlled study on the treatment of type-2 odontoid fractures in the elderly. The key results of this study will be applicable to evidence-based guidelines and benefit the growing elderly population.

Since surgery among the elderly is associated with a 4-day longer hospital stay, the results of this randomised controlled trial (RCT) will have direct implications on health policy and medical decision-making (unpublished data).

Function and HRQoL are important pillars of value-based care. Thus, this study uses a functional patient-reported questionnaire as its primary endpoint. We discarded mortality as the primary endpoint due to the fragility of patients above 70 years of age, where hazard causes overlap and sample size issues arise [43].

### Limitations

This randomised controlled study employs an open-label design, where both the investigator and the patient know which treatment they will receive. Unfortunately, the open-label design has an inherent selection bias, where patients can drop out during enrolment or give informed consent prior to inclusion in this study.

A review of the allocation concealment methods of major medical journals in 2015 found that 19% of trials involved an inadequate allocation method and 22% were without appropriate reporting of the randomisation method [44]. Our protocol uses SNOSE allocation concealment and simple randomisation. The sealed envelope technique requires good clinical practice and discipline of the part of the enrolling physician, since translucency and premature opening can corrupt the randomisation process [45]. Simple randomisation avoids deciphering block randomisation but risks different sample sizes in treatment and control groups. Transparency of the randomisation process and close supervision of the randomisation process by the principle investigator and the R&D Council will control the quality of this RCT.

One-year progression-free survival and 1-year all-cause mortality are common primary endpoints for RCTs. Due to the frailty of the elderly study participants, an overlap of an unrelated and natural cause of death with injury-related mortality causes high variation and would require a significantly greater sample size. Since HRQoL defines the value of life years gained, this study uses the NDI as the primary endpoint, with EQ-5D and survival as secondary endpoints.

We have planned to deal with dropouts for death in our sample size calculation, adopting an inflation rate. However, death will represent an important competing event. It is possible that if surgery has a favourable impact on mortality, more people will die in the control group, and earlier. These people who die might be those more compromised. This means that people in the control group who survive at 1 year might be those who are healthier, which might dilute the actual effect of the intervention on disability. To adjust for competing events we aim to use a subdistribution hazards approach to adjust for mortality [46].

The results from this study will provide overdue evidence regarding the treatment of type-2 odontoid fractures in the elderly. The QALYs gained with optimal treatment will support policy-makers and clinicians with their informed decisions.

### Trial status

This protocol been approved and registered with the Uppsala Regional Ethics Committee on 18 May 2011(no. 2011/068). It was registered retrospectively with ClinicalTrials.gov on 25 August 2015 (NCT02789774; pre-results). Patient inclusion for USOFT started in February 2012 in the Department of Surgical Sciences at Uppsala University Hospital, Uppsala, Sweden, and in October 2014 in the Department of Spine Surgery at Malmö University Hospital, Malmö, Sweden. Inclusion of other departments over the course of time is planned. Recruitment is planned to be completed before 31 December 2018.

## Additional files

Additional file 1: Standard Protocol Items: Recommendations for Interventional Trials (SPIRIT) 2013 Checklist: recommended items to address in a clinical trial protocol and related documents. (DOC 122 kb)

Additional file 2: Model informed consent form given to participants or surrogates (the original form uses the Swedish language). (PDF 60 kb)

## Abbreviations

ADL: Activities of daily living
ASA: Classification according to the American Society of Anaesthesiologists
BMI: Body Mass Index
C: Cervical vertebrae
CCI: Charlson Comorbidity Index
CI: Confidence interval
CONSORT: Consolidated Standards of Reporting Trials
CT: Computed tomography
DXA: Double x-ray absorptiometry
EQ-5D: EuroQol group questionnaire
HR: Hazard ratio
HRQoL: Health-related quality of life
ICD-10: International Classification of Diseases
ITT: Intention-to-treat
IV: Intravenous
MCID: Minimal clinically important difference
NDI: Neck Disability Index
NPR: National Patient Registry (patientregistret, Socialstyrelsen)
OR: Odds ratio
R&D: Research and development
RCT: Randomised controlled trial
RR: Risk ratio
RRR: Relative risk reduction
SAE: Serious adverse events
SCI: Spinal cord injury
SNOSE: Sequentially numbered, opaque sealed envelope
SPSS: Statistical Package for the Social Sciences
SweSpine: Swedish Spine Registry
USOFT: Uppsala Study on Odontoid Fracture Treatment
VAS: Visual Analogue Scale
