# Supplementary material for: Surgical vs. non-surgical management of displaced type-2 odontoid fractures in patients aged 75 years and older: study protocol for a randomised controlled trial
Source: Trials. 2018 Aug 22;19:452. doi: 10.1186/s13063-018-2690-8 (PMC6106890; doi:10.1186/s13063-018-2690-8)
Supplement: Supplementary file 2 — Model informed consent form given to participants or surrogates (the original form uses the Swedish language). (PDF 60 kb) [file 13063_2018_2690_MOESM2_ESM.pdf]

Patient # ___________

Information on your participation in the USOFT-trial

You suffered from a fracture of the second neck vertebra, an odontoid fracture. Due to a lack of evidence, treatment guidelines differ between hospitals in Sweden. Neck collar application and surgical stabilization are viable treatment options, which both have their strengths and limitations. Until now it is unclear which method is the better treatment for you. This study compares the results of both methods and will include up to 50 patients.

Background and purpose

A fracture of the second neck vertebra has an implicit risk of spinal cord injury as well as chronic neck pain. To counteract these complications, the neck is stabilized externally or internally for 3 months until the fracture is healed. Most common stabilization methods are neck collar or surgery. Main advantage of collar treatment is the avoidance of surgical complications, but neck immobilization may be troublesome and fracture healing is not as predictable as with surgery. Especially in elderly patients, fracture non-union is common, which has been associated with spinal cord injury and chronic pain. Other complications are pressure sores cause by the neck collar, as well as malnutrition, causing unwanted weight-loss.

During surgery, the first and the second vertebra are stabilized using a screw-construct. Disadvantages with this treatment are surgical complications, i.e. infections or thrombosis. Furthermore, the surgical stabilization of a previously mobile segment may increase neck stiffness. Main advantage with surgery are a higher rate of fracture healing, as well as the avoidance of the 3 months of semi-rigid collar treatment.

Procedure

If you accept participation in this study you will be randomised with equal chances to either the non-surgical or the surgical treatment method. Depending on the random allocation you will either receive a semi-rigid neck collar or surgery. Follow-up of both treatments are equal. You will be called to planned follow-up visits at your regional hospital, with no difference to patients outside of this study. Beyond that, you will receive surveys sent to you at four time points for one year, where you may honestly reply to questions on function and health. If you have trouble entering the information by yourself, our dedicated research nurse will be able to assist you with telephone-based interviews. Being enrolled in this study you will called to two additional radiographical investigations outside of the minimally required follow-up routine. The radiation exposure with regard to these two investigations has been judged to be of minor importance for your health.

The study protocol has been reviewed and cleared by the regional ethics committee of Uppsala.

Risks

Both non-surgical and surgical treatment arms are associated with risks. The neck collar is uncomfortable, it can cause skin lesions and discomfort especially while eating or during night. Pressure sores alongside the upper collar line are not uncommon, even though the collar has been fitted meticulously. Several patients may experience swallowing problems or

difficulties to breathe. Furthermore, personal hygiene (i.e. shaving) are complicated. After a certain time, the neck collar can start to smell, which may interfere with social activities, until the collar is changed. Neck collar treatment has a relatively higher rate of fracture non-union, with a risk of chronic pain or spinal cord affection, requiring surgical intervention months after the original injury. Even if time to fracture healing is not affected by treatment, the convalescence may appear protracted if a neck collar has to be worn for three months. It takes several weeks to regain neck muscles, which lose function after three months of collar treatment.

Surgical complications are mainly associated with the intervention itself. General anesthesia has certain risks especially for elderly people. Additionally, during surgery, important blood vessels or nerves may be injured. A surgical infection or venous thrombosis may complicate the postoperative recovery. After surgery, one can expect reduced neck rotation, which may have consequences while reversing a vehicle or while bicycling. According to our experience elderly patients are generally less troubled by the neck stiffness.

Advantages

Main advantage of non-surgical collar treatment is the avoidance of anaesthesia and surgical risks.

Advantages of surgical treatment are a shorter convalescence, and that no neck collar is required postoperatively. After approximately two weeks, as soon as the wound has healed, you are allowed to perform activities. Furthermore, surgical complications are uncommon, implying to a lower risk for fracture non-union, spinal cord injury, or chronic neck pain.

The meticulously planned follow-up, is beneficial for all study participants with shorter follow-up intervals and longer follow-up than required by the hospital's standard protocol.

Management of study results and data protection

After gathering of the study data, they are stored in a database administered by the Department of Orthopaedics, Uppsala University Hospital, Sweden. Your reported outcome measures and the study data are treated according to patient data protection legislation. Results will be presented anonymised, where individual patients are not identifiable.

Responsible for data protection according to legislation is the Department of Orthopaedics, Uppsala University Hospital, Sweden. Once every year, you are entitled to a free summary of your individual information collected during this study. You will be given the possibility to correct false information. Please call or write to one of the study contact persons if you are interested in this service.

Study results communication

The results of this study will be presented at scientific meetings and published in medical journals. If you are interested in your individual study results, or in a copy of the published study results we will be glad to provide you with this information. Please call or write to one of the study contact persons if you are interested in the results.

Compensation and liability

You are not entitled to a compensation with regard to the participation in this study. If you suffer an injury while in the Swedish health care system, you may be entitled to financial compensation under the Patient Injury Act.

Voluntary participation

Your participation is voluntary. If you choose not to participate in this study, you will receive a neck collar for 12 weeks, which is the current standard treatment at Uppsala University Hospital. Follow-up will then be performed within the current spinal fracture plan. Even if you participate in this study you are free to abort follow-up without the necessity to explain why. An abortion of the study participation will not affect how you are treated by health care providers.

Consent to participate in the USOFT-trial

The enrolling physician confirms:

The patient received both oral and written information on the Uppsala Study on Odontoid Fracture Treatment (USOFT). The patient was given the possibility to ask questions which were answered according to best available knowledge. The patient understood that enrolment in the study participation is voluntary and that he/she may abort participation without threatening his/her treatment or follow-up.

The patient consented to individual data collection, their storage, analysis and publication, if the Swedish patient data protection legislation is followed. This includes access to patient journal by study investigators.

....
Date

....
Participant signature

....
Participant name (in capital letters)

....
Enrolling physician signature

....
Enrolling physician name (in capital letters)
